# A study on the effect of detector resolution on gamma index passing rate for VMAT and IMRT QA

**DOI:** 10.1002/acm2.12285

**Published:** 2018-02-20

**Authors:** WuiAnn Woon, Paul B Ravindran, Piyasiri Ekayanake, Vikraman S, Yivonne YF Lim, Jamsari Khalid

**Affiliations:** ^1^ Department of Radiation Oncology The Brunei Cancer Center Bandar Seri Begawan Brunei Darussalam; ^2^ Faculty of Science Universiti Brunei Darussalam Bandar Seri Begawan Brunei Darussalam; ^3^ Department of Radiation Oncology Christian Medical College& Hospital Vellore India

**Keywords:** IMRT/VMAT, MLC systematic errors, sensitivity, correlation, gamma criteria, EPID, diode arrays

## Abstract

The main objectives of this study are to (1) analyze the sensitivity of various gamma index passing rates using different types of detectors having different resolutions and (2) investigate the sensitivity of various gamma criteria in intensity‐modulated radiation therapy (IMRT) and volumetrically modulated arc therapy (VMAT) quality assurance (QA) for the detection of systematic multileaf collimator (MLC) errors using an electronic portal imaging device (EPID) and planar (MapCheck2) and cylindrical (ArcCheck) diode arrays. We also evaluated whether the correlation between the gamma passing rate (%GP) and the percentage dose error (%DE) of the dose–volume histogram (DVH) metrics was affected by the finite spatial resolution of the array detectors. We deliberately simulated systematic MLC errors of 0.25 mm, 0.50 mm, 0.75 mm, and 1 mm in five clinical nasopharyngeal carcinoma cases, thus creating 40 plans with systematic MLC errors. All measurements were analyzed field by field using gamma criteria of 3%/3 mm, 3%/2 mm, 3%/1 mm, and 2%/2 mm, with a passing rate of 90% applied as the action level. Our results showed that 3%/1 mm is the most sensitive criterion for the detection of systematic MLC errors when using EPID, with the steepest slope from the best‐fit line and an area under the receiver operating characteristic (ROC) curve >0.95. With respect to the 3%/1 mm criterion, a strong correlation between %GP and %DE of the DVH metrics was observed only when using the EPID. However, with respect to the same criteria, a 0.75 mm systematic MLC error can go undetected when using MapCheck2 and ArcCheck, with an area under the ROC curve <0.75. Furthermore, a lack of correlation between %GP and %DE of the DVH metrics was observed in MapCheck2 and ArcCheck. In conclusion, low‐spatial resolution detectors can affect the results of a per‐field gamma analysis and render the analysis unable to accurately separate erroneous and non‐erroneous plans. Meeting these new sensitive criteria is expected to ensure clinically acceptable dose errors.

## 1 INTRODUCTION

Patient‐specific quality assurance (QA) for intensity‐modulated radiation therapy (IMRT) and volumetrically modulated arc therapy (VMAT) is extremely important in ensuring quality care for cancer patients in radiation therapy. Various methods, including the use of an ion chamber,^1^ two‐dimensional (2D) array detectors,^2^, ^3^ and an electronic portal imaging device (EPID),^4^, ^5^ have been employed during patient‐specific QA in pretreatment verification to detect possible errors between the dose calculated by the treatment planning system (TPS) and the measured dose. Due to the increasing complexity of modulated treatment plans and delivery, point dose measurements using an ion chamber alone may not be sufficient to verify dosimetric accuracy because a modulated plan can generate a steep dose slope near the organs at risk.

A common tool for evaluating the agreement between the calculated dose and the measured dose is the quantitative comparison of the planar dose distribution using the gamma index^6^. Task Group (TG) 119 generated by the American Association of Physicists in Medicine (AAPM) described the following acceptance criteria: a 3% dose difference (%DD) with a global normalization method and a 3‐mm distance‐to‐agreement (DTA) for a per‐field analysis. In addition, an action level of a 90% gamma passing rate (%GP) is applied with a dose threshold of 10% to remove background noise.^7^ However, many studies^8^, ^9^, ^10^, ^11^ have suggested that a lack of correlation exists between %GP and dosimetric accuracy even when more stringent gamma acceptance criteria are used.

Previous studies^8^, ^9^, ^10^, ^11^, ^12^ suggesting the insensitivity of gamma analysis have been based on similar approaches, such as (1) a per‐field analysis by reducing the acceptance criteria %DD and DTA simultaneously, for example, 3%/3 mm, 2%/2 mm, 1%/1 mm; (2) measurements made with commercial QA devices with a detector spacing of at least 7 mm; and (3) a correlation of the %GP with the percentage dose error (%DE) from a dose–volume histogram (DVH) model. The last approach uses a poor‐resolution detector on a homogeneous phantom and applies the data to a patient CT dataset to derive DVH. In addition, Bailey et al.^13^ reported that undersampling by low‐spatial resolution array detectors may potentially affect the responses of a gamma index analysis. Moreover, a recent study showed that not all induced errors can be captured by the 3DVH software^14^ and that a huge discrepancy in %DE is found on certain DVH metrics, ranging from an average value of −67.88% to 15.26% between the TPS and a COMPASS reconstructed dose,^15^ in addition to large DDs observed between the TPS and 3DVH.^12^ Furthermore, Nelms et al.^16^ showed that a major contributor to the insensitivity of gamma analysis is the DTA threshold due to modern linear accelerators that can maintain an accuracy of 1 mm using a multileaf collimator (MLC). This finding raises concern about whether the lack of a correlation between %GP and %DE will occur only on QA devices with low‐spatial resolution and a stringent acceptance criterion of only 2%/2 mm and 1%/1 mm. Although an acceptance criterion of 3%/3 mm has been reported by many authors^8^, ^9^, ^10^, ^11^, ^12^, ^16^ to be a poor predictor of dosimetric accuracy, new standardized gamma acceptance criteria for IMRT and VMAT QA have yet to be established.

Our main objective is to study the effect of detector resolution on the gamma index passing rate. This goal was achieved by investigating (1) the sensitivity of various gamma acceptance criteria by simulated MLC systematic errors in IMRT and VMAT plans; (2) the correlation between patient DVH errors reconstructed using trajectory log files and %GP; (3) the consistency, sensitivity, and performance across EPID, planar, and cylindrical diode arrays; and (4) whether the same action level and gamma criteria applied in IMRT QA can be applied in VMAT QA.

## 2 MATERIALS AND METHODS

### 2.A Patient selection and treatment planning

Five head and neck patients diagnosed with nasopharyngeal carcinoma (NPC) were selected from our database for this study. All five cases were generated with the Eclipse™ planning system (version 13, Varian Medical Systems, Palo Alto, CA, USA) and were clinically approved and treated using a nine‐field simultaneous integrated boost IMRT on a TrueBeam V. 2.0 equipped with a Millennium 120‐leaf MLC (Varian Medical Systems). To develop real‐world clinical examples, each of the clinical plans was copied and reoptimized with the same planning objectives using the dose–volume optimizer and progressive resolution optimizer (version 13.0.26, Varian Medical Systems) to generate the IMRT and VMAT plans, respectively. The final volume dose was calculated using the anisotropic analytic algorithm (version 13.0.26, Varian Medical Systems) with a grid size of 1 × 1 × 1 mm^3^.

A two‐arc VMAT and a nine‐field IMRT plans were generated using 6 MV photon beams with a 600 MU min^−1^ dose rate and the following prescription: 70 Gy (2 Gy/fraction) to the planning target volume (PTV) containing a primary gross tumor and gross positive lymph nodes, a 63 Gy (1.8 Gy/fraction) to the PTV with high‐risk nodes, and a 56 Gy (1.6 Gy/fraction) to the PTV with low‐risk nodes. When planning a risk volume, a 5‐mm margin was added around critical organs such as the spinal cord and brainstem to account for the geometric uncertainties of an organ and thereby achieve maximum doses of <45 Gy and <54 Gy, respectively. Many other normal structures, such as the parotid glands (left‐L, right‐R), the mandibular and temporal mandibular joints, and the optic chiasm and the optic nerves, were included in the optimization process; however, only the parotids, spinal cord, brainstem, and the PTV receiving 70 Gy (PTV_70_) were analyzed in this study. For all NPC plans, at least 98% of the PTVs must be achieved with 95% of the prescription dose, not exceeding more than 107% of the prescription dose.

### 2.B Simulation of MLC errors

All copied IMRT and VMAT plans were exported in DICOM format from the TPS to an external computer operating customized Python software (The Scientific Python Development Environment, V. 2.7+, The Spyder Development Team, http://www.Python.org/). Each field in the IMRT and VMAT plans consisted of 166 and 177 control points, respectively. Each control point contained information on all MLC leaves position; therefore, beam apertures change shape in a discrete manner from one control point to the next. MLC errors were simulated in all control points in every field using the program such that both MLCs were systematically perturbed and resulted in an opening of MLC apertures by 0.25 mm, 0.50 mm, 0.75 mm, and 1 mm, except for the actual delivery, which consisted of random errors. To study random errors, an additional trajectory log file is necessary for plan modification. The trajectory log file has a binary format and records the actual performance of the machine and dosimetric parameters such as the MLC, the gantry position, the dose rate, and the jaw position during treatment delivery. All modified plans were imported back into the TPS for recalculation of the dose distribution, and the DVH changes due to simulated MLC systematic errors were analyzed.

### 2.C Dose evaluation in DVH‐based metrics

To evaluate the DD in each DVH metric, all of the modified plans were compared with the original plan, and the %DE was subsequently calculated using the following equation:

$$\text{Dose Error}\;\left(\%\right)=\frac{D_{\mathrm{modified}}\left(\text{cGy}\right)-D_{\mathrm{Original}}\left(\text{cGy}\right)}{D_{\mathrm{Original}}\left(\text{cGy}\right)}\times 100$$

where D_modified_ is the DVH dose calculated for each structure from the plans with the systematic MLC errors and the actual dose calculated from the plans with the actual MLC position from the log file. D_original_ is the DVH dose calculated for each structure, with the original plan as a reference.

The relative %DE was calculated for each structure as shown in Table 1. In addition, the D_mean_ of PTV70, R parotid, and L parotid and the D_2%_ of the spinal cord and brainstem were evaluated.

**Table 1:** The average changes in the %DE for the DVH metrics due to MLC errors

Percentage dose difference of DVH metrics between the recalculated and the original plans (%)						
Treatment	MLC errors	Structure				
		Brainstem D_2%_	Spine D_2%_	R parotid D_Mean_	L parotid D_Mean_	PTV_70_ D_Mean_
IMRT	Random	0.05 ± 0.06	0.02 ± 0.12	0.00 ± 0.01	0.00 ± 0.02	0.41 ± 0.95
	0.25 mm	2.62 ± 0.71	2.27 ± 0.32	2.67 ± 0.19	2.39 ± 0.39	1.47 ± 0.29
	0.50 mm	5.24 ± 1.37	4.58 ± 0.60	5.33 ± 0.39	4.77 ± 0.77	2.92 ± 0.58
	0.75 mm	7.95 ± 2.10	6.93 ± 0.86	7.99 ± 0.59	7.14 ± 1.15	4.36 ± 0.87
	1.00 mm	10.66 ± 2.83	9.33 ± 1.03	10.65 ± 0.79	9.52 ± 1.54	5.80 ± 1.16
VMAT	Random	−0.24 ± 0.69	−0.63 ± 0.49	1.03 ± 0.39	−0.70 ± 0.25	0.19 ± 0.07
	0.25 mm	1.46 ± 0.64	0.81 ± 0.30	0.97 ± 0.22	1.05 ± 0.39	0.78 ± 0.23
	0.50 mm	2.98 ± 1.32	1.60 ± 0.49	1.95 ± 0.46	2.09 ± 0.72	1.39 ± 0.41
	0.75 mm	4.52 ± 1.99	2.40 ± 0.70	2.94 ± 0.69	3.14 ± 1.05	1.99 ± 0.59
	1.00 mm	6.05 ± 2.66	3.30 ± 0.84	3.92 ± 0.93	4.19 ± 1.39	2.60 ± 0.78

### 2.D Detectors and software for dose evaluation

All IMRT plans were delivered for pretreatment verification and measured using the EPID (Varian Medical Systems) and MapCheck2 (Sun Nuclear Corporation, Melbourne, FL, USA), while the EPID and ArcCheck (Sun Nuclear Corporation) were used for dosimetric verification in all VMAT plans. The EPID system used was an aS1000 amorphous silicon portal imager with a resolution of 0.392 mm and a measuring area of 40 × 30 cm^2^. The MapCheck2 has a measuring area of 26 × 32 cm^2^ that consists of 1527 solid‐state SunPoint^®^ diode detectors with a resolution of 0.8 × 0.8 mm, a diagonal detector spacing of 7.07 mm and 1 cm parallel detector spacing. The ArcCheck dimensions are 21 cm in length and 21 cm in diameter, consisting of 1386 solid‐state SunPoint^®^ diode detectors with a resolution of 0.8 × 0.8 mm and a detector spacing of 1 cm.

Portal Dosimetry (version 13, Varian Medical Systems) was used to compare the measured dose distribution and predicted dose distribution generated from the TPS using the portal dose imager prediction algorithm (version 13.0.26, Varian Medical Systems). All measurements conducted using the MapCheck2 and ArcCheck were compared with a calculated dose distribution generated by the TPS, and these values were analyzed with SNC Patient™ v. 6.6.2 (Sun Nuclear Corporation).

### 2.E Gamma analysis

An absolute gamma analysis was performed in the IMRT and VMAT plans, and a relative gamma analysis was also included for the VMAT plans. Global dose normalization with four different acceptance criteria (3%/3 mm, 3%/2 mm, 3%/1 mm, and 2%/2 mm) were applied in all analyses with a dose threshold of 10% to remove the noise. An action level of 90% for %GP was established in our institute for the IMRT and VMAT as per the AAPM TG‐119 protocol.^7^ All measurements were conducted after the completion of the array and absolute dose calibration according to the manufacturer's specifications.

### 2.F Consistency, correlation, and sensitivity analysis

Pearson's correlation coefficient (*r*) was used to statistically analyze the relationship between %GP and %DE. Moreover, a *P*‐value <0.05 was necessary to conclude that the variables were correlated. An *r* value of 0–0.39 was regarded as a weak correlation between %GP and %DE, 0.4–0.59 as moderate, 0.6–0.79 as strong, and 0.8–1 as very strong.

Linear regression was used to compute the best‐fit line from a plot of the %GP vs MLC errors. The slope of the best‐fit line was used to evaluate the sensitivity of each gamma criterion. The *R* ^2^ value from a linear regression was also used to study how well the %GP could explain the changes in the %DE due to MLC errors. Furthermore, the sensitivity of the various acceptance criteria and performance of each QA device were assessed with a receiver operating characteristic (ROC) curve and the area under the ROC curve (AUC). An AUC close to 1 indicates that the acceptance criteria for a certain QA device could accurately differentiate an error plan with at least a 3% DD from a plan with no errors.

Furthermore, the consistency of the %GP generated from three different QA devices with respect to simulated MLC systematic errors of the same magnitude was assessed for the five different NPC cases. In addition, the statistical correlation (r), R^2^, and the slope of the best‐fit line were included when evaluating the consistency of three different QA devices.

### 2.G True errors and true error positions

Forward IMRT planning using a single field generated such that 20% of the prescription dose was delivered to a field size of 10 × 8 cm^2^ while simultaneously boosting a 0.5 × 8 cm^2^ gap to 80% of the prescription dose. In addition, systematic errors of 0.5 mm, 1 mm, 5 mm, and 10 mm were simulated in 16 pairs of alternating MLC leaves resulted in an opening of MLC apertures. The simple forward IMRT planning was used to study the effect of the resolution of different detector systems (i.e., EPID, MapCheck2 and ArcCheck) in detecting true errors and their actual position. To assess whether the EPID, MapCheck2, and ArcCheck can actually detect these simulated errors located at the right positions, a 2D vs 2D comparison between the measured dose and calculated dose generated from the original plan with and without simulated MLC errors was performed. Furthermore, gamma analysis with the same acceptance criteria previously described was also used to assess whether the %GP could correctly include these simulated errors when different detectors were compared.

## 3 RESULTS

### 3.A True errors and true position

**Figure 1:**
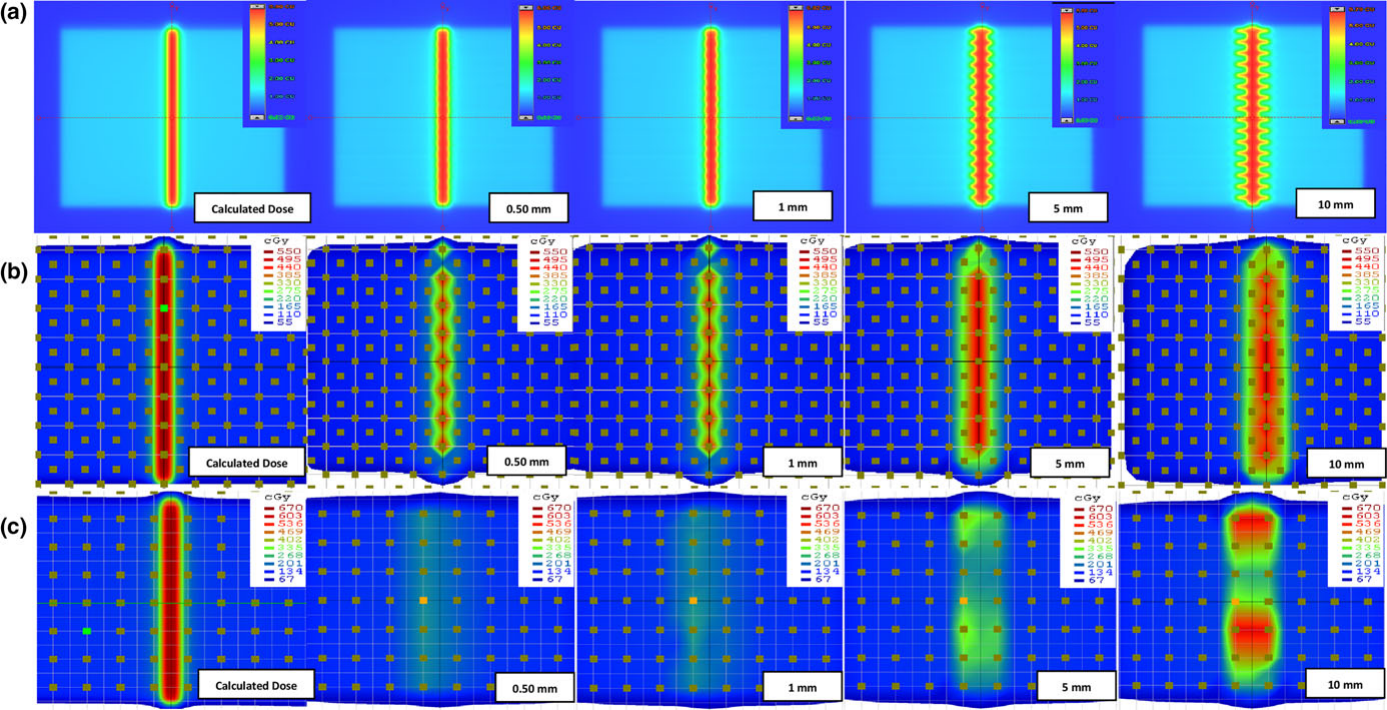
Resolution influence of different detector system for the detection of a systematic MLC error. The results of measured dose using the EPID are shown in (a), while those performed on MapCheck2 and ArcCheck are shown in (b) and (c), respectively.

**Figure 2:**
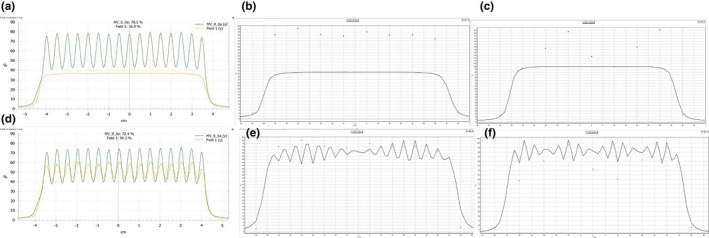
Resolution influence of different detector system for the detection of quantitative errors and its true position due to 5 mm of MLC systematic errors. Dose profile comparison between the calculated plan without simulated MLC errors and the measured dose with simulated errors through *y*‐axis from the EPID are shown in (a), while those from MapcCheck2 and ArcCheck are shown in (b) and (c), respectively. In addition, dose profile comparison between calculated plan and measured dose with simulated MLC errors through *y*‐axis from the EPID are shown in (d), while those from MapcCheck2 and ArcCheck are shown in (e) and (f), respectively.

**Figure 3:**
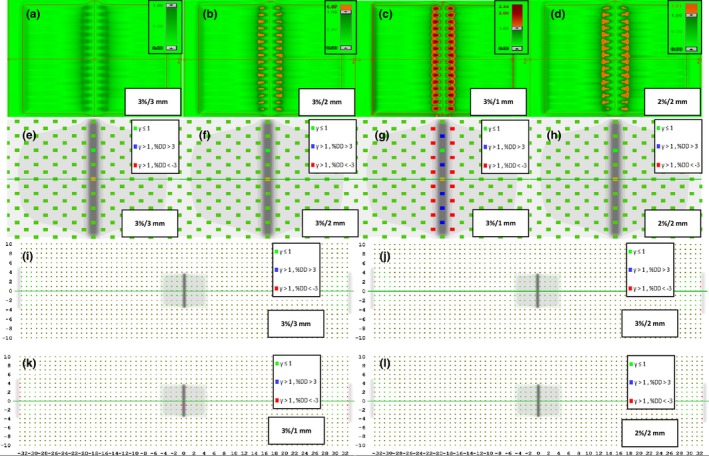
Resolution influence of different detector systems as 5 mm of the systematic MLC error is evaluated. The results of gamma passing and failing points based on acceptance criteria of 3%/3 mm, 3%/2 mm, 3%/1 mm, and 2%/2 mm performed on the EPID are shown in (a)–(d), respectively, while those performed on MapCheck2 are shown in (e)–(h) and those performed on ArcCheck are shown in (i)–(l).

Planar doses measured by the EPID, MapCheck2, and ArcCheck were compared, and the effects of detector resolution on dose distribution are illustrated in Fig. 1. In the case of the EPID, all simulated MLC errors in the measured doses/measured dose distributions are certainly noticeable compared with those in the calculated doses/calculated dose distributions. In contrast, simulated MLC errors greater than 0.5 mm were not distinguishable in measured doses by the MapCheck2 due to the inferior resolution caused by the large diode spacing relative to the resolution of the EPID. The error detection was deteriorated in the ArcCheck due to the larger diode spacing. Examples of 5 mm systematic MLC errors were used for further analysis, as this is the minimal detectable error shown in the ArcCheck results (Fig. 1); in addition, more accurate quantitative errors and the actual positions of the simulated MLC errors can only be observed in y profiles using the EPID, as shown in Fig. 2. Five millimeter systematic MLC errors were not detected in any QA device when a conventional 3%/3 mm gamma index was used, as shown in Fig. 3; the same results were observed in the case of the MapCheck2 when 3%/2 mm and 2%/2 mm gamma indices were applied. Gamma evaluation showed (Fig. 3(c)) that 3%/1 mm is the most sensitive criterion when using the EPID because it can be observed that the gamma failing points increase in the position where MLC errors were simulated, as indicated by the red‐shaded region. For the MapCheck2 with acceptance criteria of 3%/1 mm, the gamma failing points correctly included not only the systematic MLC errors but also wrong errors, as indicated by the blue points; this effect resulted in artificially lower passing rates with an increase in false‐positive rates. By applying more stringent gamma criteria such as 3%/2 mm, 3%/1 mm, and 2%/2 mm when using the ArcCheck, gamma failing points increased mostly near the exit diodes. It should be noted that an increase in the false‐negative rate was observed because all detected MLC errors do not present the true errors. Furthermore, we evaluated the gamma analysis results by applying even more stringent criteria such as 1%/1 mm, which has not been previously described, to determine whether all simulated MLC errors can be detected when using the ArcCheck. However, such errors are not due to systematic MLC errors in the true position, although lower passing rates are obtained by including wrong errors.

### 3.B Sensitivity evaluation of various gamma criteria

#### 3.B.1 EPID

**Figure 4:**
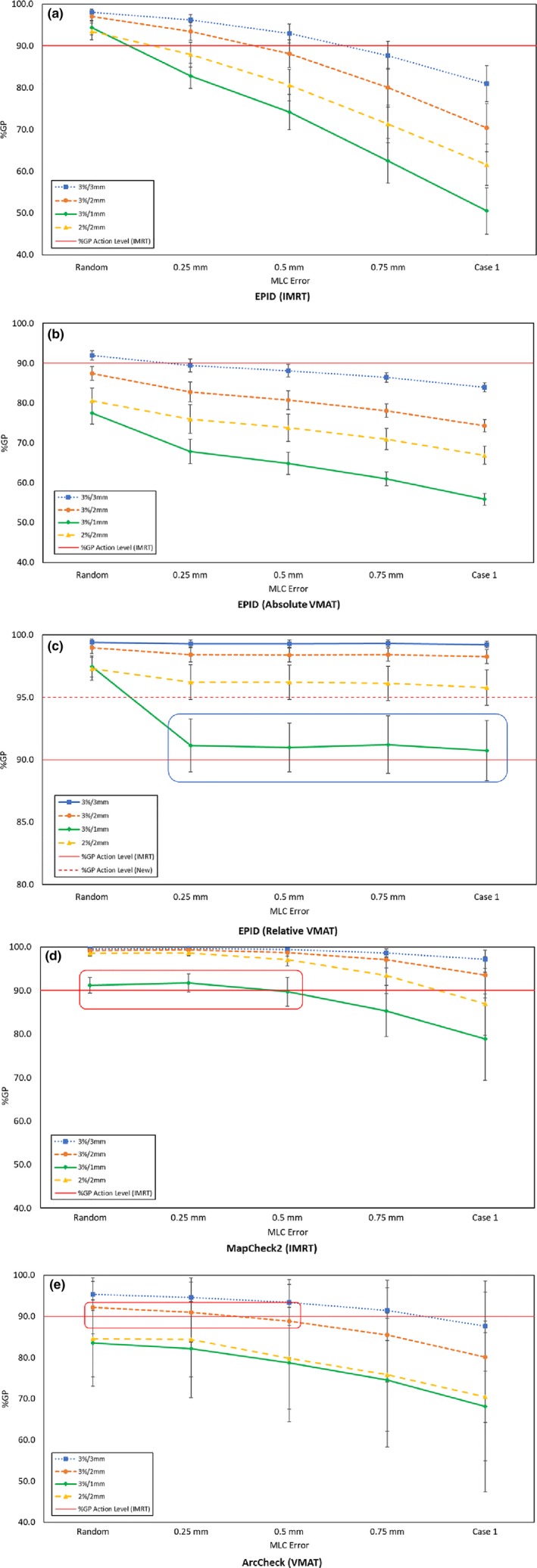
Sensitivity of the various gamma criteria for the detection of a systematic MLC error. The results of the absolute gamma analyses performed on the IMRT QA using the EPID are shown in (a), while those performed on the VMAT QA are shown in (b), and the relative gamma analyses are shown in (c). The results of the absolute gamma analyses performed on the IMRT QA using MapCheck2 are shown in (d), while those on the VMAT QA using ArcCheck are shown in (e).

An EPID was used to evaluate the sensitivity of various gamma criteria based on the slope of the best‐fit line, as illustrated in Fig. 4. A higher negative slope of the best‐fit line indicated greater sensitivity of a given gamma criterion for systematic MLC errors, and the results indicated that 3%/1 mm is the most sensitive criterion in the EPID for the detection of a systematic MLC error for both the IMRT and the VMAT plans. However, verification of VMAT plans using absolute gamma comparison with 3%/1 mm failed to achieve a passing rate of 90%. In contrast, verification of the VMAT plans using a relative gamma comparison with 3%/1 mm was less sensitive, as indicated by a lower negative slope than that for the absolute gamma method. Moreover, the passing rate was much higher than 90%, even when a 1‐mm systematic MLC error was considered. When a 95% passing rate was applied as a new action level for 3%/1 mm using the relative gamma method, a 0.25‐mm systematic MLC error could be detected.

#### 3.B.2 MapCheck2

Table 2 shows that the most sensitive gamma criterion from the MapCheck2 for the IMRT plans was 3%/1 mm, except for case 4, for which it was 2%/2 mm. Fig. 4 shows that a systematic MLC error of up to 0.5 mm was not detected with a gamma criterion of 3%/1 mm. A false‐negative result was observed, indicating that a 90% passing rate as the action level could not distinguish between the original and erroneous plans.

**Table 2:** Slope of the best‐fit line between %GP of various gamma criteria and MLC errors

Slope of the best‐fit line							
Treatment	QA device	Method	Case	Acceptance criteria			
				3%/3 mm	3%/2 mm	3%/1 mm	2%/2 mm
IMRT	EPID	Absolute gamma analysis	1	−5.15	−7.84	−12.10	−8.80
			2	−4.69	−7.15	−11.16	−8.80
			3	−4.63	−7.07	−11.12	−8.03
			4	−4.25	−6.72	−10.61	−7.77
			5	−2.60	−4.52	−8.99	−6.74
VMAT	EPID	Absolute gamma analysis	1	−2.14	−3.44	−5.44	−3.69
			2	−1.70	−2.64	−3.81	−2.54
			3	−2.07	−3.44	−5.61	−3.67
			4	−1.75	−2.81	−4.79	−3.05
			5	−1.91	−3.13	−5.42	−3.22
VMAT	EPID	Relative gamma analysis	1	−0.01	−0.14	−1.64	−0.42
			2	−0.01	−0.09	−0.73	−0.20
			3	−0.05	−0.19	−1.85	−0.42
			4	−0.07	−0.19	−1.46	−0.36
			5	−0.02	−0.11	−1.03	−0.17
IMRT	MapCheck2	Absolute gamma analysis	1	−1.34	−2.83	−6.36	−5.52
			2	−0.30	−0.77	−2.10	−1.68
			3	−0.73	−1.71	−4.70	−3.28
			4	−0.53	−1.41	−1.30	−3.08
			5	0.01	−0.06	−1.06	−0.57
VMAT	ArcCheck	Absolute gamma analysis	1	−3.78	−5.33	−6.33	−4.87
			2	−0.28	−0.55	−1.08	−1.28
			3	−3.64	−5.57	−6.60	−5.92
			4	−0.64	−1.17	−2.04	−2.96
			5	−1.06	−2.21	−3.18	−3.38

#### 3.B.3 ArcCheck

Table 2 shows that the most sensitive gamma criterion with the ArcCheck for cases 1 and 3 was 3%/1 mm, and the most sensitive criterion was 2%/2 mm for all other cases. With respect to the most sensitive criteria of 3%1 mm and 2%/2 mm, Fig. 4 shows that all VMAT plans had a passing rate of less than 90%. Systematic MLC errors of up to 0.75 mm were not detected with a gamma criterion of 3%/2 mm when a >90% passing rate for the original plan was considered.

### 3.C Sensitivity and performance of various gamma criteria based on ROC analysis

**Figure 5:**
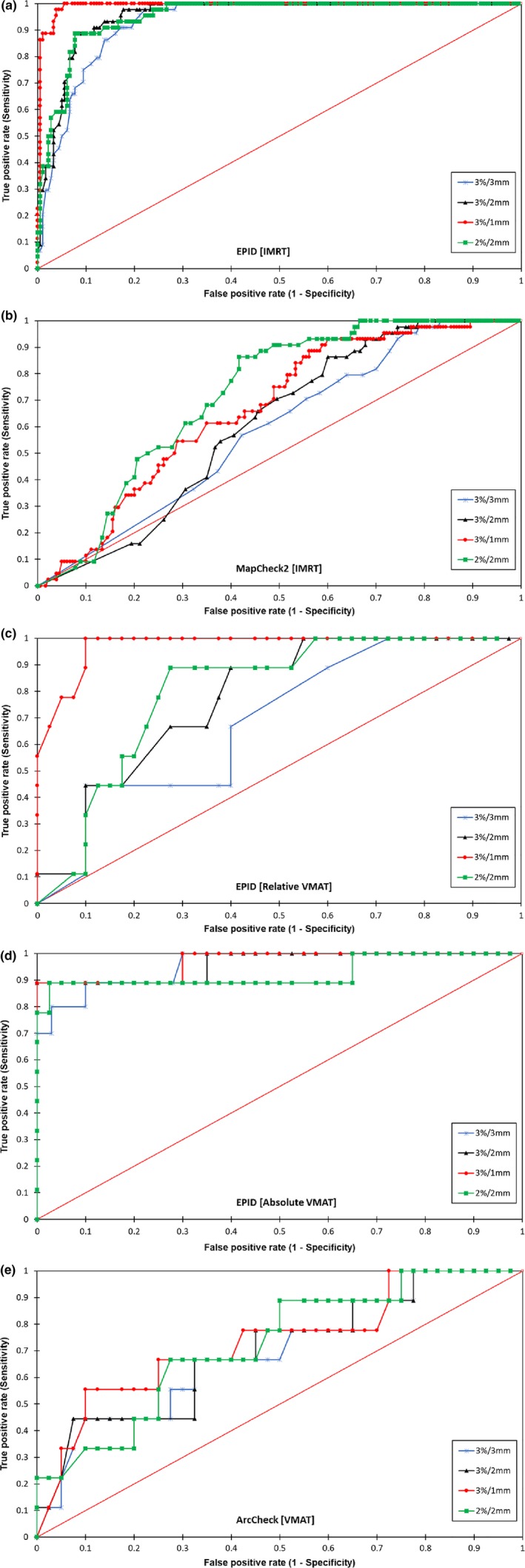
Comparison of the ROC curves for the various gamma criteria when using the EPID, MapCheck2, and ArcCheck. The results of a comparison of the ROC curves for the various gamma criteria applied on the IMRT QA when using the EPID are shown in (a), MapCheck2 (b), and on the VMAT QA when relative and absolute gamma analyses are performed when using the EPID are shown in (c) and (d), respectively. The ROC curves for the various gamma criteria when using ArcCheck are shown in (e).

Further analysis of the sensitivity and performance of the various acceptance criteria for each QA device with an ROC is shown in Fig. 5, and the AUC is shown in Table 3. The most sensitive criterion for the IMRT and VMAT QA using the EPID was again 3%/1 mm, and an AUC >0.95 indicated excellent performance in predicting the %DE from the %GP. However, 2%/2 mm was the most sensitive criterion for the MapCheck2 and ArcCheck and achieved an AUC of <0.75, which indicated poor accuracy in predicting the %DE from the %GP.

**Table 3:** A comparison of the AUC values for various gamma criteria for different QA devices

AUC [standard error, 95% confidence interval]						
Treatment	QA device	Method	Acceptance criteria			
			3%/3 mm	3%/2 mm	3%/1 mm	2%/2 mm
IMRT	EPID	Absolute gamma analysis	0.930 [0.016, 0.898–0.962]	0.952 [0.013, 0.927–0.978]	0.992 [0.005, 0.982–1]	0.950 [0.014, 0.923–0.977]
VMAT	EPID	Absolute gamma analysis	0.958 [0.031, 0.897–1]	0.965 [0.036, 0.895–1]	0.970 [0.031, 0.910–1]	0.933 [0.065, 0.805–1]
VMAT	EPID	Relative gamma analysis	0.629 [0.101, 0.431–0.826]	0.740 [0.078, 0.588–0.892]	0.966 [0.022, 0.923–1]	0.766 [0.074, 0.620–0.912]
IMRT	MapCheck2	Absolute gamma analysis	0.590 [0.042, 0.505–0.667]	0.610 [0.039, 0.532–0.686]	0.669 [0.040, 0.589–0.745]	0.723 [0.035, 0.655–0.792]
VMAT	ArcCheck	Absolute gamma analysis	0.694 [0.105, 0.442–0.853]	0.701 [0.105, 0.448–0.860]	0.735 [0.106, 0.476–0.892]	0.722 [0.101, 0.473–0.867]

### 3.D Changes in the DE% with respect to the MLC error

**Figure 6:**
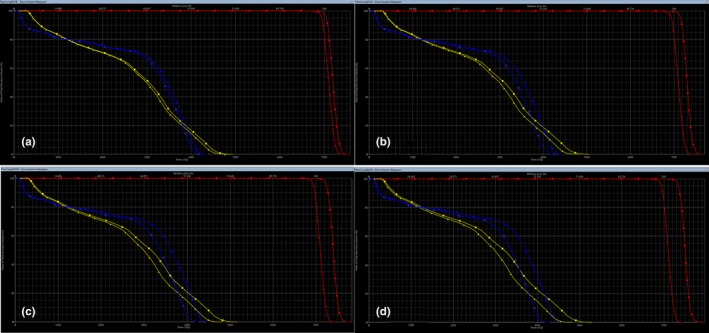
DVH comparison between original plan (triangles) and modified plan (squares) with simulated MLC systematic errors. DVH comparison between original plan and modified plan with systematic MLC error of 0.25 mm are shown in (a), 0.50 mm (b), 0.75 mm (c), and 1 mm are shown in (d). Blue: spinal cord; yellow: brainstem; red: planning target volume.

Table 1 shows the relative %DE values for the original plan and the modified plan edited with log files referred to as “Random.” Table 1 and Fig. 6 also show the relative %DE between the original plan and the modified plan edited with systematic MLC errors of different magnitudes. The relative %DE values between the original and the modified plan with a random error were well within a 3% DD for both the IMRT and VMAT plans. In addition, an increase in the magnitude of the simulated MLC systematic errors caused the average relative %DE of the D_2%_ of the brainstem and the spinal cord and the average relative %DE of the D_mean_ for PTV_70_, L parotid and R parotid to increase.

### 3.E Statistical correlation between %GP and %DE

**Figure 7:**
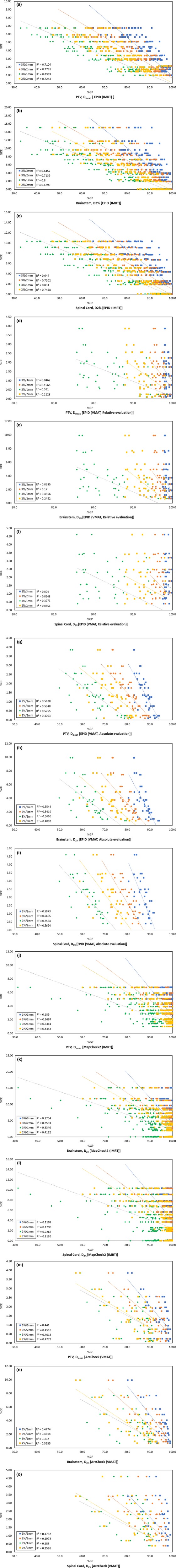
Correlation between the %GP of the various gamma criteria and the %DE. The correlation between the %GP of the gamma criteria of 3%/3 mm, 3%/2 mm, 3%/1 mm, and 2%/2 mm and the %DE for the D_mean_ of PTV and the D_2%_ of the spinal cord and the brainstem are shown in (a)–(c) for absolute gamma analyses performed on the IMRT QA using the EPID, while the relative and absolute gamma analyses performed on the VMAT QA are shown in (d)–(f) and (g)–(i), respectively. The results of the absolute gamma analyses performed on the IMRT QA using MapCheck2 are shown in (j)–(l), while those on the VMAT QA using ArcCheck are shown in (m)–(o).

The statistical correlations (*R* ^2^ and *r*) between %DE and %GP with their respective *P*‐values are shown in Fig. 7 and Table 4. The most sensitive acceptance criterion of 3%/1 mm for the pretreatment verification using the EPID shows a better correlation between the %GP and the relative %DE with respect to each structure than the other acceptance criteria. However, the correlation between the %GP and the relative %DE with respect to each DVH metric from the ArcCheck and MapCheck2 was better with 2%/2 mm, indicating that the sensitivity of the various acceptance criteria differs in certain cases.

**Table 4:** Correlation between the %GP of various gamma criteria and the %DE for the DVH metrics

Pearson's correlation coefficient ®													
Treatment	QA devices	Methods	Acceptance criteria	Brainstem, D_2%_		Spine, D_2%_		R parotid, D_Mean_		L parotid, D_Mean_		PTV _70_, D_Mean_	
				*r*	*P*	*r*	*P*	*r*	*P*	*r*	*P*	*r*	*P*
IMRT	EPID	Absolute gamma analysis	3%/3 mm	−0.80	<0.001	−0.80	<0.001	−0.80	<0.001	−0.77	<0.001	−0.84	<0.001
			3%/2 mm	−0.85	<0.001	−0.85	<0.001	−0.85	<0.001	−0.82	<0.001	−0.88	<0.001
			3%/1 mm	−0.89	<0.001	−0.91	<0.001	−0.92	<0.001	−0.89	<0.001	−0.92	<0.001
			2%/2 mm	−0.82	<0.001	−0.86	<0.001	−0.85	<0.001	−0.80	<0.001	−0.85	<0.001
VMAT	EPID	Absolute gamma analysis	3%/3 mm	−0.75	<0.001	−0.77	<0.001	−0.74	<0.001	−0.77	<0.001	−0.75	<0.001
			3%/2 mm	−0.74	<0.001	−0.81	<0.001	−0.71	<0.001	−0.75	<0.001	−0.73	<0.001
			3%/1 mm	−0.75	<0.001	−0.87	<0.001	−0.71	<0.001	−0.78	<0.001	−0.76	<0.001
			2%/2 mm	−0.63	<0.001	−0.75	<0.001	−0.62	<0.001	−0.63	<0.001	−0.61	<0.001
VMAT	EPID	Relative gamma analysis	3%/3 mm	−0.25	0.077	−0.06	0.66	−0.19	0.19	−0.24	0.09	−0.21	0.13
			3%/2 mm	−0.41	<0.05	−0.23	0.10	−0.33	<0.05	−0.43	<0.05	−0.40	<0.05
			3%/1 mm	−0.68	<0.001	−0.57	<0.001	−0.51	<0.001	−0.68	<0.001	−0.62	<0.001
			2%/2 mm	−0.50	<0.001	−0.26	0.07	−0.41	<0.001	−0.50	<0.001	−0.46	<0.001
IMRT	MapCheck2	Absolute gamma analysis	3%/3 mm	−0.41	<0.001	−0.35	<0.001	−0.39	<0.001	−0.40	<0.001	−0.44	<0.001
			3%/2 mm	−0.50	<0.001	−0.42	<0.001	−0.46	<0.001	−0.47	<0.001	−0.52	<0.001
			3%/1 mm	−0.58	<0.001	−0.48	<0.001	−0.52	<0.001	−0.52	<0.001	−0.58	<0.001
			2%/2 mm	−0.64	<0.001	−0.56	<0.001	−0.61	<0.001	−0.62	<0.001	−0.67	<0.001
VMAT	ArcCheck	Absolute gamma analysis	3%/3 mm	−0.69	0.077	−0.42	<0.05	−0.61	<0.001	−0.58	<0.001	−0.66	<0.001
			3%/2 mm	−0.69	<0.05	−0.44	<0.05	−0.61	<0.001	−0.59	<0.001	−0.67	<0.001
			3%/1 mm	−0.63	<0.001	−0.43	<0.05	−0.57	<0.001	−0.55	<0.001	−0.63	<0.001
			2%/2 mm	−0.74	<0.001	−0.51	<0.001	−0.64	<0.001	−0.61	<0.001	−0.69	<0.001

### 3.F Consistency analysis of different QA tools

Pretreatment verification of the IMRT and VMAT plans with the EPID is more consistent than verification with MapCheck2 and ArcCheck, as shown in Fig. 4. An acceptance criterion of 3%/1 mm was the most sensitive for all plans, with simulated MLC systematic errors of similar magnitude. In addition, Fig. 7 and Table 4 indicate that the acceptance criterion 3%/1 mm consistently showed the highest correlation between the %GP and the %DE in a per‐field analysis. Furthermore, a systematic MLC error of 0.25 mm when using the EPID can be consistently detected with passing rates of 90% and 95% applied as the action levels for the IMRT and VMAT gamma analyses, respectively.

## 4 DISCUSSION

In this study, the resolution effect of different detector systems on the gamma index passing rate was investigated. The results suggest that 3%/1 mm is the most sensitive gamma criterion for the detection of a systematic MLC error when performing IMRT and VMAT QA using the EPID. In contrast, the MapCheck2 and ArcCheck do not show consistent performance when analyzing the slope of the best‐fit line; our results indicate that either 3%/1 mm or 2%/2 mm is the most sensitive gamma criterion when a systematic MLC error of the same magnitude is simulated.

A 90% passing rate as the action level for IMRT QA described by AAPM TG 119 is relevant when used with a more stringent criterion of 3%/1 mm with the EPID. As indicated by our results, a sudden drop in the passing rate can identify an erroneous plan. Finally, a relatively strong correlation between the %GP and the %DE for all IMRT QA performed using the EPID was observed, which has not been previously reported.

Using the most sensitive criterion for the MapCheck2 with a 90% passing rate as the action level for the IMRT QA, false positives and negatives occurred, and a passing rate below 90% did not indicate large differences in the DVH and vice versa. Furthermore, a weak correlation was observed between the %GP and the %DE for all of the IMRT QA performed with the MapCheck2. These results are similar to previously reported results.^9^, ^12^, ^15^

The ArcCheck displayed the worst performance among all three devices as a QA tool. As shown in Fig. 1, the device failed to detect a simulated MLC error of 1 mm in the IMRT plan. Furthermore, a reasonable action level could not be established when a more stringent criterion was considered. The original plan had already failed to achieve a passing rate higher than 90% with respect to the most sensitive gamma criteria used. Similar to the MapCheck2 results, false‐positive and false‐negative errors were also observed with the ArcCheck; the red box in Fig. 4 indicates the inability of the MapCheck2 and ArcCheck to distinguish between an original and an erroneous plan, which suggests that low‐spatial resolution affects the gamma index analysis because the dose distribution was undersampled,^17^ as confirmed by the ROC and AUC results. Worse yet is the result for certain cases in which a 0.75 mm systematic MLC error was undetected due to the poor resolution of the detectors, which could result in expected maximum doses of >54 Gy and >45 Gy in the brainstem and spinal cord, respectively. Furthermore, no consistent relationship can be established between the sensitivity of the gamma criteria derived from using the slope of the best‐fit line and a statistical correlation of the %GP and the %DE.

A strong correlation was observed between the %GP and the %DE when performing the VMAT QA using the EPID and an absolute gamma analysis. However, the original plan did not achieve a 90% passing rate; therefore, a relative gamma analysis was used instead. The passing rate in the relative gamma analysis was higher than in the absolute gamma analysis because the average DD between the calculated and measured dose distributions was minimized. This condition weakened the correlation between the %GP and the %DE (as indicated by the blue box in Fig. 4) and rendered the technique unable to detect the erroneous condition at a 90% passing rate applied as the action level. However, when a 95% passing rate with relative gamma analysis was used instead for the VMAT QA using the EPID with a weak‐to‐moderate correlation between the %GP and the %DE, a clear distinction could be drawn between the original and the erroneous plan.

It was also investigated whether the MapCheck2 and ArcCheck could produce correctly detected errors at the points where the true errors occurred in this study. As shown in Fig. 3, when the device resolution is not appropriate for detecting errors with the conventional gamma index of 3%/3 mm, the achieved gamma passing rates can lead to misleading QA results. It thus becomes increasingly important to select an appropriate device that has sufficiently high resolution, particularly when evaluating highly complex IMRT and VMAT plans such as head and neck cases, to detect errors at high‐dose gradients between targets and critical organs.

The question may still remain regarding whether a binary pass or fail classifier in a per‐field analysis can indicate the location and magnitude of a DE, but if the correct acceptance criteria are employed, 2–3% changes in the DVH metrics can be detected using a reasonable action level. Furthermore, our results are consistent with those of the study by Nelms et al.,^16^ which showed that the DTA threshold is one of the primary insensitive metrics for the gamma criteria for detecting systematic errors. One of the main limitations of this study was the limited number of patients used to investigate whether the established action levels and acceptance criteria were consistent; however, this is a pilot study, and more samples will be included in future studies. The results of this study indicate that an acceptance criterion of 3%/1 mm is the most sensitive for IMRT and VMAT QA to detect any systematic MLC errors; however, the criteria may vary between detector systems with different resolutions. Therefore, it is important to evaluate a system's limitations with respect to its detectable error range, uncertainty, and reliability before deciding on a more sensitive gamma criterion. In addition, care should be taken when establishing the action level, as this level may vary due to differences in TPS commissioning and the QA devices employed.

## 5 CONCLUSION

This study investigated the sensitivity of various gamma criteria for the detection of changes in the DVH by deliberately introducing systematic MLC errors of the same magnitude into all IMRT and VMAT plans. The correlation between the %DE and the %GP evaluated by different QA devices was also investigated. Our findings confirmed that the lack of correlation between the %DE and the %GP was due to the resolution, which was not sufficient to detect MLC systematic errors when using array detectors. This analysis suggested that detector resolution can affect gamma analysis and lead to misleading IMRT/VMAT QA results by incorrectly detecting MLC systematic errors. Our study showed that an acceptance criterion of 3%/1 mm is the most sensitive and can distinguish the original condition from an erroneous condition with a systematic MLC error using the EPID. A strong correlation between the %GP and the %DE was observed when QA was performed on a high‐resolution device such as the EPID using a gamma criterion of 3%/1 mm. Moreover, an acceptance criterion of 3%/1 mm can be applied to both the IMRT and VMAT QA; however, the action levels for the IMRT and VMAT are slightly different. The adoption of a more sensitive criterion can ensure that a plan is clinically acceptable with no systematic MLC errors when every field passes the gamma criterion.

## CONFLICT OF INTEREST

The authors declare no conflict of interest.
